# 11 cm Haughton D left cervical aortic arch aneurysm

**DOI:** 10.1186/1749-8090-8-108

**Published:** 2013-04-23

**Authors:** Pankaj Kaul

**Affiliations:** 1 Leeds General Infirmary, Yorkshire Heart Centre, Great George Street, Leeds LS1 3EX, UK

## Abstract

A 56 year old Caucasian man presented with sudden loss of consciousness while driving and was found to have an 11 cm Haughton D type left cervical aortic arch aneurysm with normal brachiocephalic branching and normal descending thoracic laterality but with considerable tortuosity and redundancy of aortic arch. The aneurysm arose between the left common carotid artery and the left subclavian artery. It compressed and stretched the left common carotid artery, compressed the pulmonary trunk and the left pulmonary artery, stretched the vagus, left recurrent laryngeal and left phrenic nerves and caused extreme deviation of trachea, severely compromising the tracheal lumen. Patient underwent successful interposition graft replacement of distal aortic arch under total circulatory arrest and selective unihemispherical cerebral perfusion.

## Background

Left cervical aortic arch is a rare developmental anomaly of aorta and only around 70 cases are described in world literature. It is further classified into five distinct types on the basis of brachiocephalic branching, arch and descending aortic laterality and redundancy of the transverse aorta. Left cervical aortic arch, in general, and Haughton D type, in particular, is prone to aneurysm formation due to abnormal flow patterns and tortuosity and redundancy of aorta.

The unique features about the surgical anatomy and presentation of this patient include the origin of the aneurysm from the aortic arch in the narrow segment between the left common carotid and the left subclavian arteries with extreme displacement of both. There was a separation of more than three inches between the origins of left common carotid artery and the left subclavian artery. There was extreme tortuosity and redundancy of the distal aortic arch and disproportionate vertical enlargement of the aneurysm. A number of anatomically diverse structures, including trachea, left common carotid artery, left subclavian artery and main and left pulmonary artery, left vagus and phrenic nerves, had been compressed, displaced and distorted. The aneurysm did not rupture despite reaching a size of eleven cms.

## Case presentation

**Figure 1:**
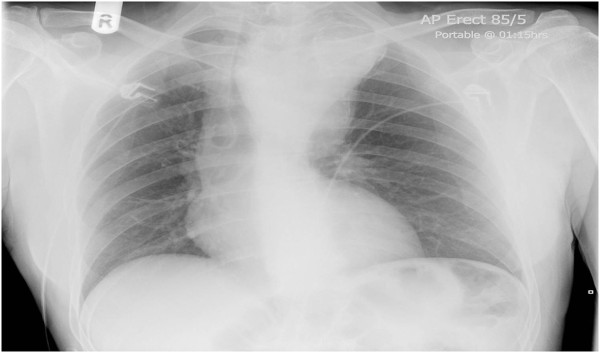
Chest X-ray showing a large superior mediastinal shadow suggestive of arch aneurysm extending well into neck with extreme tracheal deviation.

**Figure 2:**
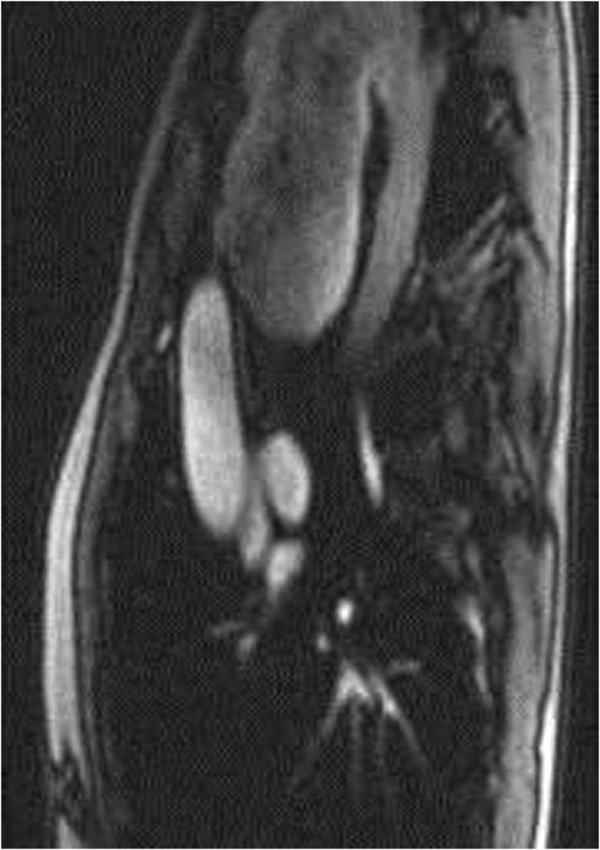
MR scan showing massive arch aneurysm.

**Figure 3:**
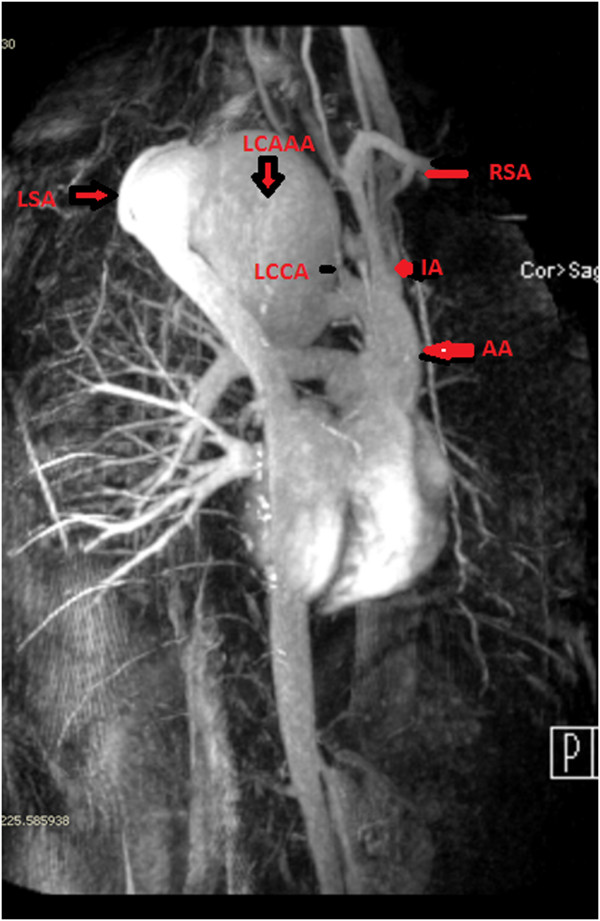
**MR scan demonstrating massive left cervical aortic arch aneurysm (LCAAA) arch aneurysm arising between the origins of left common carotid artery (LCCA) and left subclavian artery (LSA).** LCCA is displaced anteriorly and LSA posteroinferiorly into left chest. Also note the redundancy of distal arch with more than 7.5 cm separation between LCCA and LSA. Ascending aorta (AA) is normal-sized and the right subclavian artery (RSA) arises normally.

A 56 year old man had transient loss of consciousness while driving. His wife pulled over and, in A& E, he was found to have a large superior mediastinal shadow, extending into neck, on chest X-ray (Figure 1). CT scan revealed a 7 cm × 11 cm saccular aneurysm arising from the distal aortic arch between the origins of the left common carotid and left subclavian arteries, with cephalic ectopy. Transthoracic echocardiogram demonstrated large saccular aneurysm of the distal aortic arch with turbulent flow at the left subclavian artery. MR scan confirmed the presence of a large distal aortic arch aneurysm arising from the aortic arch between the origins of left common carotid and left subclavian arteries with extreme displacement of both vessels, the left common carotid artery being displaced anteriorly and to the right and also stretched and distorted owing to vertical expansion of the aneurysm posterior to it, and the left subclavian artery displaced well down and posteriorly into the left chest with even greater distortion (Figures 2 and 3). An aortogram clarified the situation further by showing that the aneurysm involved the arch beyond the origin of the left common carotid artery and that the left subclavian artery had been displaced by the aneurysm posteroinferiorly into the left chest. There was a great tortuosity and redundancy of the aorta between the two vessels and the aorta and aneurysm rose well into the neck. Coronary angiogram was normal.

**Figure 4:**
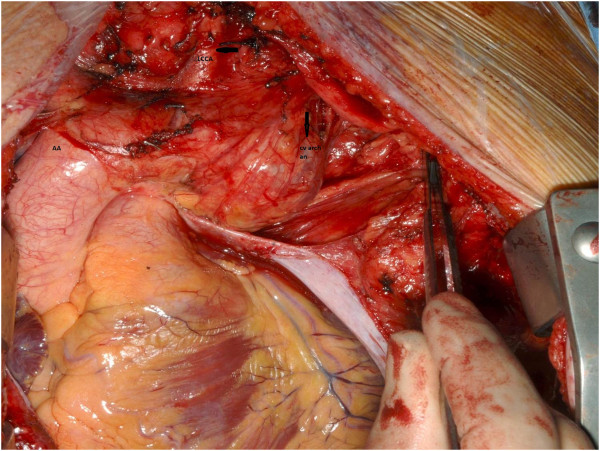
**Intraoperative photograph shows large left cervical aortic arch aneurysm (cv arch an) arising beyond the left common carotid artery (LCCA), extending partly behind the left common carotid artery, with distal limit of the aneurysm not clearly visible from the median sternotomy incision.** Ascending aorta (AA) is normal.

**Figure 5:**
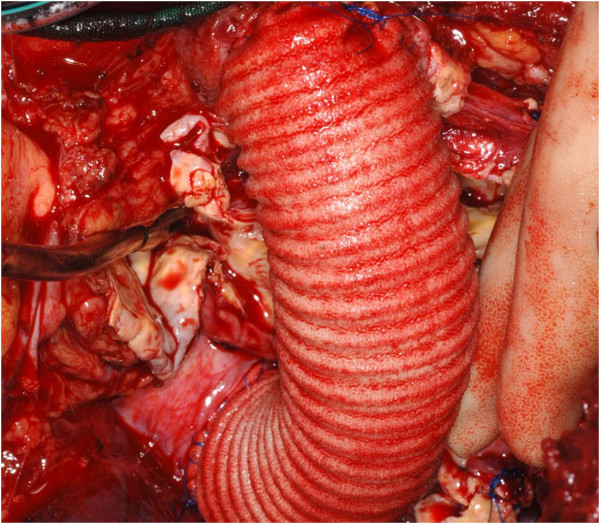
The continuity of the arch and descending thoracic aorta re-established by a 30 mm Vascutex Dacron graft.

At median sternotomy, the distal arch rose into the neck for a variable distance. There was a 9 cm × 11 cm aortic arch aneurysm arising distal to the left common carotid artery and proximal to the left subclavian artery, extending in both vertical and horizontal directions, with tortuosity and redundancy of the distal arch, causing compression, deviation and distortion of a number of adjacent structures (Figure 4). Trachea was severely compressed with deviation to the right and the aneurysm formed a posterior relation of the ascending aorta and displaced it and the left common carotid artery anteriorly. The left pulmonary artery and the main pulmonary trunk were displaced posteriorly and inferiorly and the left vagus and the left phrenic nerves were stretched over the entire length of the aneurysm. Ascending aorta and proximal arch were normal sized. The proximal descending aorta was diffusely atheromatous. In order to get a better access to the descending aorta, a T shaped anterolateral extension was made in the 4th space. Cardiopulmonary bypass was instituted with common femoral arterial and right atrial venous cannulations and patient cooled to 14°C. Left ventricle was vented through right superior pulmonary vein. Under lower body deep hypothermic circulatory arrest (LBDHCA) and selective antegrade cerebral perfusion (SACP), the arch aneurysm was excised and a 30 mm VASCUTEK interposition graft used to establish the continuity between the arch distal to the left common carotid artery and the proximal part of the descending aorta (Figure 5). The left subclavian artery was ligated and not reimplanted as it was severely atheromatous along its entire length. Selective antegrade cerebral perfusion was achieved through external cannulation of the left common carotid artery by a 10 F MEDTRONIC perfusion cannula using IL/min flow.

**Figure 6:**
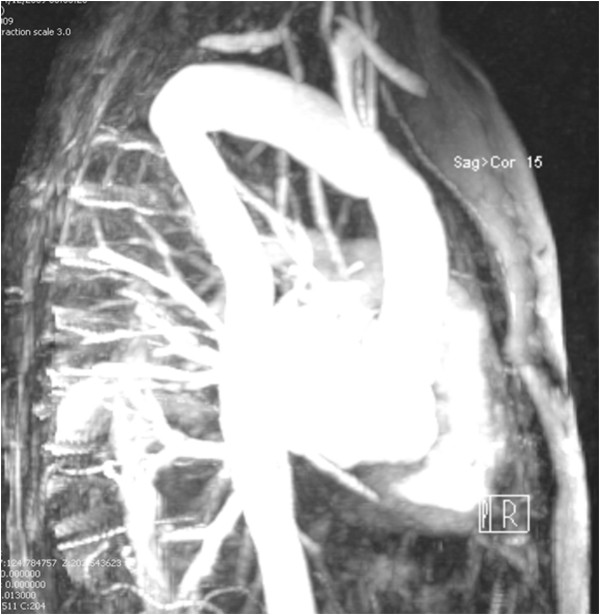
A postoperative MR scan showing satisfactory repair.

Patient was extubated on first postoperative day and, except for mild hoarseness of voice which improved, made uncomplicated postoperative recovery. Biopsy of aortic tissue showed a picture consistent with arteriosclerosis and he was discharged home on seventh postoperative day. Follow up MR scan 4 months later showed satisfactory repair (Figure 6) although he needed thyroplasty with a titanium clip for left vocal cord palsy. He continues to be fully active and asymptomatic 4 years after surgery and his follow up MR scans are normal.

## Discussion

Cervical aortic arch (CAA) is a rare congenital anomaly consisting of cephalic displacement of aortic arch. Haughton described five distinct forms based on configuration of the aorta, sequence of brachiocephalic branching and embryogenesis. Type A has separate external and internal carotid artery branches from the aortic arch. Type B has dual common carotid arteries. Type C is a left cervical arch with right sided descending aorta and bicarotid trunk. Type D has normal brachiocephalic branching, redundant transverse aorta and left-sided descending aorta. Type E is a right cervical aortic arch with a right descending aorta and an aberrant left subclavian artery [1].

Embryologically, cervical aortic arch provides a link in the embryogenesis of the great arteries of thorax. The cervical location is attributed to the persistence of the right or left third arch instead of the fourth arch, thereby rerouting the aorta via the neck [2]. However, failure of the normal caudal migration of the fully developed fourth arch from the occipital level in the thoracic cage at the seventh week of gestation has also been advocated as the reason for cephalad ectopy of the arch [3]. D’Cruz et al. believed CAA to be the result of a confluence of the third and fourth arches as a result of the anomalous growth of the pharyngeal pouch tissue, along with failure of the normal fourth arch to descend into the thorax with heart [4]. Finally, development of 2nd branchial arch into the aortic arch with persistence of first arch on the side involved as the proximal internal carotid artery has been advocated as the embryological basis. [5].

Left cervical aortic arch (LCAA) occurs as an isolated anomaly and is usually silent. Presenting features vary from a pulsatile swelling in the supraclavicular fossa to respiratory symptoms. The predominant congenital heart anomalies associated with left cervical aortic arch include patent ductus arteriosus, tetralogy of Fallot and pseudotruncus [6]. Association with deletion of chromosome 22q 11 has been described. There are less than 70 reports of left cervical arch in world literature yet aneurysmal degeneration in such an ectopic, tortuous, meandering and redundant vessel is not uncommon. Premature atherosclerosis due to abnormal shear stresses generated by the cervical arch is the commonest cause. When complicated by aneurysmal degeneration, symptoms are not unlike other aortic arch aneurysms. Dysphagia, stridor, wheezing, coughing and apneic spells, aspiration, swallowing problems, syncope, weakened arm or leg pulses and subclavian steal have all been reported [2].

Only a few cases of Haughton D type left cervical aortic arch (LCAA) aneurysms have been described. Takahashi et al. described a LCAA aneurysm between left common carotid artery (LCCA) and left subclavian artery (LSA), complicated by tracheal stenosis, managed by total arch replacement via median sternotomy with bilateral supraclavicular extensions, with good relief of tracheal compression [7]. Mitsoumori reported interposition graft replacement of transverse aorta between IA and LSA and reconstruction of LCCA in a 38 year old female with LCAA aneurysm between LCCA and LSA with persistent left SVC, under median sternotomy, total circulatory arrest and innominate artery selective cerebral perfusion (SCP) [8]. Noguchi et al. repaired another Haughton D type LCAA aneurysm in a 59 year old man with a dacron interposition graft from just beyond LCCA to the beginning of distal transverse aorta (DTA) with branch reconstruction of LSA, under total circulatory arrest (TCA) [9]. Tsukamoto et al. described repair of LCAA aneurysm with LSA aneurysm and coarctation [10] and Imai et al. reported reconstruction with interposition graft of LCAA with coarctation [11]. Hirao et al. reported LCAA aneurysm which had been thought to be a mediastinal tumour [12], and Higuchi employed left thoracotomy and partial cardiopulmonary bypass through descending aorta and IVC to repair LCAA aneurysm just distal to LCCA with aberrant and tortuous LSA [13].

Our patient had a 9 × 11 cm aneurysm arising distal to LCCA. The transverse arch extended for a variable distance into the neck, and the aneurysm extended from the root of neck into the upper left hemithorax. The LSA was tortuous, aberrant and severely calcified in its intrathoracic course and arose from the upper descending thoracic aorta. The arch and proximal descending aorta were severely atheromatous and the transverse arch between the LCCA and LSA was redundant and severely aneurysmal. The arch aneurysm distorted all the adjoining structures and extended superiorly, inferiorly and laterally, compressing pulmonary trunk and LPA, compressing and pushing trachea to the right, and stretching ascending aorta and the LCCA in front of it. Patient’s presentation with a fainting spell could have been due to obstruction of LCCA or left subclavian steal. Total circulatory arrest(TCA) with hemispherical selective cerebral perfusion (SCP), as opposed to partial CPB with two clamps, was used because anastomosis needed to be done quite close to the LCCA and the transverse aorta was diffusely atheromatous. Hemispherical SCP through left common carotid artery (LCCA) was adequate as seen by copious returns from IA and LSA.

The unique features about our patient, in contrast to a few reports previously published, include the massive expansion of the small segment of aorta between the left common carotid artery and the left subclavian artery into an 11 cm aneurysm with its apex in the neck. There was a separation of more than three inches between the origins of left common carotid artery and the left subclavian artery with extreme displacement of both, severe tortuosity and redundancy of the distal aortic arch and disproportionate vertical enlargement of the aneurysm. A number of anatomically diverse structures, including trachea, left common carotid artery, left subclavian artery, main and left pulmonary artery, left vagus and phrenic nerves, had been distorterd, displaced and compressed and yet, despite reaching a size of eleven cms, the aneurysm did not rupture.

## Conclusions

Left cervical aortic arch is a rare developmental anomaly of aorta, characterised by cephalad displacement of the aortic arch. Only around 70 cases are described in world literature. It is further classified into five distinct types on the basis of brachiocephalic branching, arch and descending aortic laterality and redundancy of the transverse aorta. Haughton described five distinct forms based on configuration of the aorta, sequence of brachiocephalic branching and embryogenesis. Type A has separate external and internal carotid artery branches from the aortic arch. Type B has dual common carotid arteries. Type C is a left cervical arch with right sided descending aorta and bicarotid trunk. Type D has normal brachiocephalic branching, redundant transverse aorta and left-sided descending aorta. Type E is a right cervical aortic arch with a right descending aorta and an aberrant left subclavian artery Left cervical aortic arch, in general, and Haughton D type, in particular, is prone to aneurysm formation due to abnormal flow patterns and tortuosity and redundancy of aorta.

The unique features about our patient, in contrast to a few reports previously published, include the massive expansion of the small segment of aorta between the left common carotid artery and the left subclavian artery into an 11 cm aneurysm with its apex in the neck. There was a separation of more than three inches between the origins of left common carotid artery and the left subclavian artery with extreme displacement of both, severe tortuosity and redundancy of the distal aortic arch and disproportionate vertical enlargement of the aneurysm. A number of anatomically diverse structures, including trachea, left common carotid artery, left subclavian artery, main and left pulmonary artery, left vagus and phrenic nerves, had been distorterd, displaced and compressed and yet, despite reaching a size of eleven cms, the aneurysm did not rupture.

## Consent

Written informed consent was obtained from the patient for the publication of this report and the accompanying images.

## Abbreviations

LBDHCA: Lower body deep hypothermic circulatory arrest; CAA: Cervical aortic arch; LCCA: Left cervical aortic arch; LCAAA: Left cervical aortic arch aneurysm; LCCA: Left common carotid artery; LSA: Left subclavian artery; SCP: Selective cerebral perfusion; DTA: Distal transverse aorta; TCA: Total circulatory arrest; IA: Innominate artery; CPB: Cardiopulmonary bypass.

## Competing interests

The author declares that he has no competing interests.
